# Effects of Pyrolysis Temperature and Chemical Modification on the Adsorption of Cd and As(V) by Biochar Derived from *Pteris vittata*

**DOI:** 10.3390/ijerph19095226

**Published:** 2022-04-25

**Authors:** Kazuki Sugawara, Kouhei Ichio, Yumiko Ichikawa, Hitoshi Ogawa, Seiichi Suzuki

**Affiliations:** 1 Faculty of Science and Technology, Seikei University, Tokyo 1808633, Japan; biolab2105@outlook.jp (K.I.); s91603@cc.seikei.ac.jp (Y.I.); seiichi@st.seikei.ac.jp (S.S.); 2 Faculty of Engineering, Tokyo University of Science, Tokyo 1258585, Japan; 3 Research Institute, Tamagawa University, Tokyo 1948610, Japan; hn@wta.att.ne.jp

**Keywords:** biochar, phytoremediation, arsenic, cadmium

## Abstract

Phytoremediation can be applied successfully to solve the serious worldwide issue of arsenic (As) and cadmium (Cd) pollution. However, the treatment of biomass containing toxic elements after remediation is a challenge. In this study, we investigated the effective use of biomass resources by converting the As hyperaccumulator *P. vittata* into biochar to adsorb toxic elements. Plant biomass containing As was calcined at 600, 800, and 1200 °C, and its surface structure and adsorption performances for As(V) and Cd were evaluated. Pyrolysis at 1200 °C increased the specific surface area of the biochar, but it did not significantly affect its adsorption capacity for toxic elements. The calcined biochar had very high adsorption capacities of 90% and 95% for As(V) and Cd, respectively, adsorbing 6000 mmol/g-biochar for As(V) and 4000 mmol/g-biochar for Cd. The As(V) adsorption rate was improved by FeCl_3_ treatment. However, the adsorption capacity for Cd was not significantly affected by the NaOH treatment. In conclusion, it was found that after phytoremediation using *P. vittata* biomass, it can be effectively used as an environmental purification material by conversion to biochar. Furthermore, chemical modification with FeCl_3_ improves the biochar’s adsorption performance.

## 1. Introduction

Environmental pollution caused by anthropogenic and natural sources is a serious problem. Soil and water pollution caused by toxic elements, especially arsenic (As) and cadmium (Cd), can have grave consequences on human health. As is ubiquitous in the environment, including in the atmosphere, rocks, water, and organic matter [1,2]. Such health hazards caused by naturally occurring As have been reported in many Southeast Asian countries. Argos et al. estimated that tens of millions of people in Bangladesh and India suffer from health hazards caused by orally ingested As from drinking water and consuming food [3]. As ingestion can cause acute symptoms such as vomiting, diarrhea, muscle spasms, dysphagia, skin erosion, and death after coma [4]. As a chronic disease, inflammation of the mucous membranes of the eyes, nose, and throat is observed, and as the disease progresses, skin disorders such as black pigmentation and depigmentation keratosis appear, and the disease becomes more serious causing peripheral neuritis and cancer [4]. 

Cd is released into the environment by the mining of zinc and other metals, and health hazards are often reported in river basins adjacent to these mines and smelters [5]. Many cases of health hazards caused by Cd have been confirmed in Asia, and soil and water contamination have been reported in Thailand [6] and China [7]. Acute illnesses caused by Cd include nausea and abdominal pain. Chronic diseases reported include tubular disorders, osteomalacia, and Itai-itai disease [8]. Therefore, soil and water pollution caused by these toxic elements is a health hazard and an environmental problem that needs to be addressed promptly. To address these pollution problems, we focused on phytoremediation, an environmental remediation method using plants.

Phytoremediation is a method used for the removal of toxic substances from soil and groundwater using the absorption capacities of plants [9]. In particular, research on methods using hyperaccumulating plants, which are plants that accumulate specific elements at high concentrations [10], is being actively conducted using *Pteris vittata* L. [11], *Thlaspi caerulescens* [12], and *Alyssum lesbiacum* [13], which accumulate As, Cd, and nickel (Ni), respectively. The advantages of phytoremediation include lower cost than treatment using heavy machinery, lower environmental burden because it does not involve modification of the surrounding environment and applicability to a wide range of lands [14]. However, while valuable metals such as Ni can be reused after accumulation [15], the use of biomass with high concentrations of other toxic elements has not been studied, and incineration is the only option. Therefore, in this study, the conversion of biomass into biochar was conceived as a method to utilize the biomass after phytoremediation.

Biochar refers to the carbonized material produced by heating biomass at 250 °C or higher under oxygen-free or oxygen-limited conditions [16]. An important feature of biochar is that it possesses certain organic carbons, called fused aromatic ring structures, which are similar to charcoal. These structures are formed during pyrolysis and are key to the properties related to mineralization and adsorption. In addition, biochar has a large specific surface area and high cation exchange capacity and is stable in the environment because of its resistance to degradation [17]. Because of these characteristics, biochar is an effective adsorbent for the restoration of environments contaminated by various substances, including heavy metals. It has been reported that biochar derived from *Ginkgo biloba* adsorbed up to 93.22 mg/L of Pb(II) and 22.58 mg/L of Cu(II) at an initial concentration of 100 mg/L [18]. Oil-palm empty fruit-bunch derived biochar and rice husk derived biochar adsorbed a maximum of 18.9 mg of As(III) and 19.3 mg of As(V) per unit weight in a solution containing an initial concentration of 50 mg/L [19]. Rapeseed straw-derived biochar adsorbed 32.74 mg/g of Cd(II) in 40 mL of CdCl(II) solution with an initial concentration of 1.25 g/L [20]. Furthermore, these studies have reported that the chemical modification of biochar can improve the adsorption of toxic elements. Thus, pyrolysis temperature and post-pyrolysis chemical treatment of biochar have been shown to improve heavy metal adsorption capacity, indicating that they are promising environmental purification materials. However, there are no reports on the use of hyperaccumulators as purification materials.

In this study, biochars of As-accumulated *P. vittata* biomass were prepared and their heavy metal adsorption properties for As and Cd were evaluated to effectively utilize them after their initial use for environmental remediation. *P. vittata* absorbs As(V) through, the body in the form of As(III) at high concentrations of more than 2% of its dry weight [11]. As in the body of *P. vittata* is present as inorganic arsenic, and its localization in vacuoles and cell walls has been suggested by previous studies [21,22]. Inorganic As is known to evaporate at temperatures below 200 °C [23]. Therefore, the As that was accumulated in the biomass during the biochar production process evaporates, and it can be recovered relatively easily with a filtration-type dust collector commonly used in incinerators, to obtain clean biochar. In addition, the biochars were chemically modified with FeCl_3_ and NaOH to improve their heavy metal adsorption capacities.

## 2. Materials and Methods

### 2.1. Pyrolysis of Biochar

*P. vittata* plants were purchased from FUJITA Co. Tokyo, Japan. The raw material for biochar was *P. vittata* were grown in a field trial in Miyagi Prefecture. The As concentration in *P. vittata* has been confirmed to be approximately 20 mg/kg [24]. All field experiments comply with local and national regulations. The harvested *P. vittata* was dried naturally and divided into leaves and stems. Then, only the leaves were packed in stainless steel containers and subjected to pyrolysis in an electric furnace under an N_2_ atmosphere. During the pyrolysis process in the electric furnace, the temperature was increased to below 150 °C for 3 h, from 150 to 600 °C at 100 °C/h, and from 600 °C and above at 200 °C/h. When the desired temperature was reached, it was maintained for 5 min to complete pyrolysis. The biochar was calcined at final pyrolysis temperatures of 600, 800, and 1200 °C, and the obtained calcined biochars were used in the experiments.

### 2.2. Chemical Modifications with FeCl_3_ and NaOH

The calcined biochars were chemically modified using FeCl_3_ and NaOH. Chemical modification with FeCl_3_ was performed according to Sadegh-Zadeh and Seh-Bardan [19]. 1000 mg/L FeCl_3_ solution adjusted to pH 6 using NaOH and HCl was prepared. As a pretreatment, 7 g of biochar was finely ground until more than 90% of it passed through a 1 mm sieve. The crushed biochar was added to the FeCl_3_ solution and stirred with a magnetic stirrer for 24 h. After stirring, the solution containing the biochar was suction-filtered, and the resulting FeCl_3_ modified biochar was washed with Milli-Q water to remove the unreacted FeCl_3_. Then, it was dried in a dryer at 60 °C for 24 h. 

The procedure for chemical modification with NaOH was based on the method described by Li et al. [20]. First, 2 M NaOH solution was prepared. As a pre-treatment, 7 g of biochar from leaves was finely ground until more than 90% of it passed through a 1 mm sieve. After grinding, the biochar was added to the NaOH solution and stirred vigorously for 12 h in a hot stirrer set at 100 °C with a magnetic stirrer for modification. After stirring, the solution containing the biochar was filtered by suction. The resulting NaOH-modified biochar was washed three times with Milli-Q water and three times with 0.01 M NaHCO_3_. Then, it was dried in a dryer at 60 °C for 24 h.

### 2.3. Evaluation of Physical Properties of Biochars

A total of 9 types of biochar, including biochar calcined at each temperature (3 types) and chemically modified with FeCl_3_ and NaOH (3 types × 2 conditions) from biochar calcined at each temperature, were observed for surface structure and measured for their specific surface area. Structural observation by SEM (JSM-6360, JEOL, Tokyo, Japan) was performed on the fractions of the samples that passed through a 1 mm sieve to evaluate the differences in structure due to pyrolysis temperature and chemical modification.

A vacuum superheating pretreatment system (BELPREP-vac III, Microtrac Bell, York, PA, USA) and a specific surface area measurement system (BELSORP-mini II, Microtrac Bell, PA, USA) were used to measure the specific surface area. The sample biochars were crushed into small pieces using a mortar, set in the BELPREP-vac III, and heated at 130 °C for 3 h in a vacuum to remove water and other substances adsorbed on the sample. After pretreatment, the sample was set in a BELSORP-mini II, and the specific surface area was measured using the nitrogen adsorption method. The BET method was used to analyze the specific surface area of the obtained adsorption/desorption isotherms [25].

### 2.4. Evaluation of As and Cd Adsorption Capacities of Biochars

Nine types of biochars were evaluated for their ability to adsorb As(V) or Cd. Biochars with different pyrolysis temperatures (3 types) without chemical modification were tested for As(V) and Cd adsorption. Samples with FeCl_3_ chemical modification on biochar at different pyrolysis temperatures (3 types) were tested for As(V) adsorption only, and those chemically modified with NaOH (3 types) were evaluated only for Cd. 

The biochar samples were finely ground until more than 90% passed through a 1 mm sieve. After grinding, the samples were dried at 115 °C for 3 h to remove moisture. After drying, the samples were placed in a desiccator and allowed to cool for 24 h. Then, three empty 50 mL centrifuge tubes were prepared for each sample. In each centrifuge tube, 50 mL of As(V) (Na_2_HAsO_4_·7H_2_O) or Cd (CdCl_2_) solution was prepared at concentrations of 1, 10, 100, 150, 250, 500, and 1000 mg/L. A quantity of 0.1 g of pretreated biochar was added to each centrifuge tube. The centrifuge tubes were sealed and shaken for 24 h at 100 rpm in a shaker at 25 °C with a lateral shaking width of 4–5 cm. After shaking, the supernatant was collected from the centrifuge tube and diluted to 10 mL with Milli-Q water by adding nitric acid to achieve a sample final concentration of 5% (*v*/*v*). The test solution was then filtered through a 0.45 μm membrane filter and used as the sample for measurement. The As and Cd concentrations in the test solution were then quantified using high-frequency, inductively coupled, plasma atomic emission spectrometry (iCAP6000, Thermo Fisher Scientific, Waltham, MA, USA). The summarized experimental conditions are shown in Table 1. Adsorption tests for As(V) and Cd were also conducted using commercially available activated carbon (KD-GW-200, ASONE, Osaka, Japan) for comparison, following the same procedure.

### 2.5. Data Analysis

The obtained measurement data were checked for significant differences using ANOVA using the statistical analysis software R (version 4.0.2, R Development Core Team).

## 3. Results

### 3.1. Evaluation of Surface Structure and Specific Surface Area of Biochar

**Figure 1:**
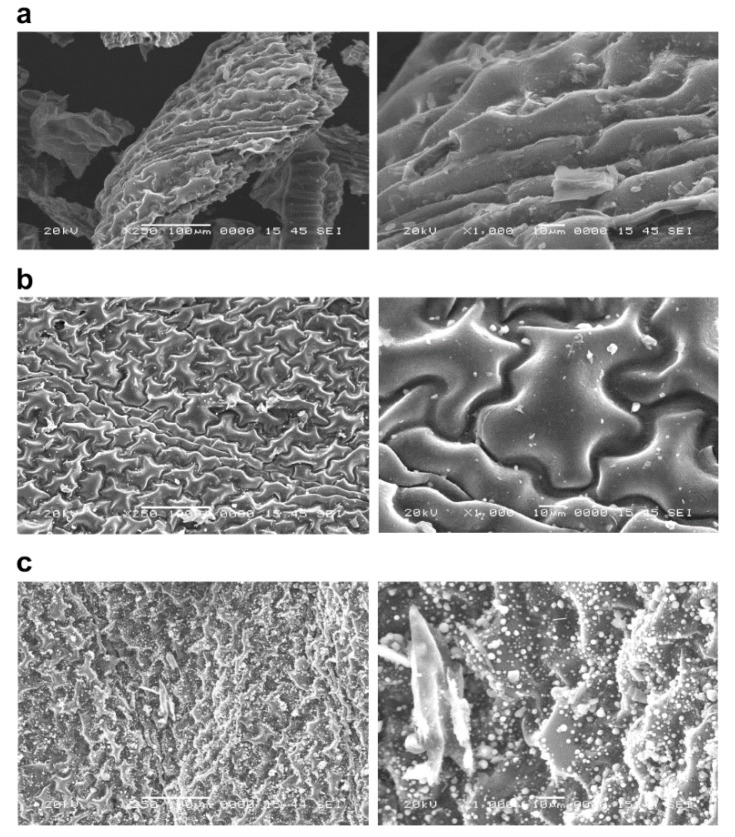
The surface structure of *P. vittata* biochar by SEM observation. Left column: Magnification 250×; Right column: Magnification 1000×; (**a**) Pyrolysis temperature: 600 °C; (**b**) Pyrolysis temperature: 800 °C; (**c**) Pyrolysis temperature: 1200 °C.

**Figure 2:**
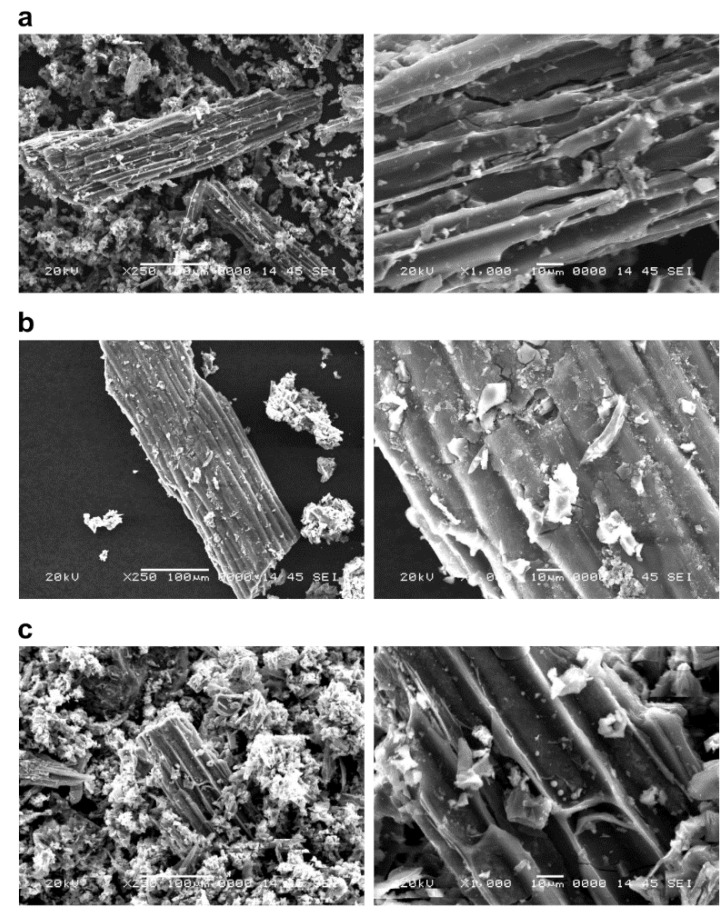
The surface structure of FeCl_3_-modified *P. vittata* biochar by SEM observation. Left column: Magnification 250×; Right column: Magnification 1000×; (**a**) Pyrolysis temperature: 600 °C; (**b**) Pyrolysis temperature: 800 °C; (**c**): Pyrolysis temperature: 1200 °C.

**Figure 3:**
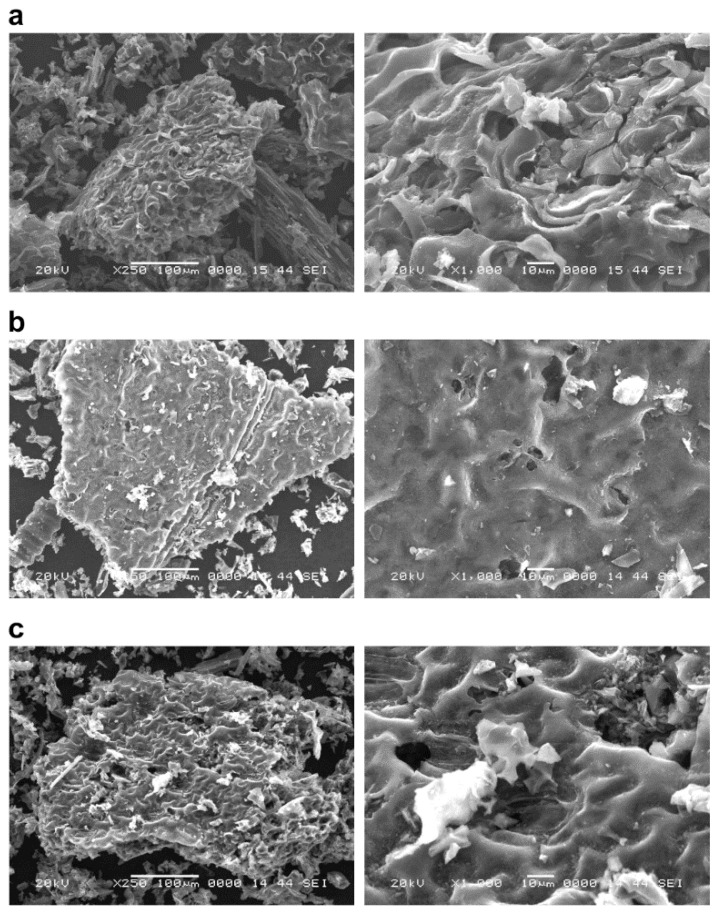
The surface structure of NaOH-modified *P. vittata* biochar by SEM observation. Left column: Magnification 250×; Right column: Magnification 1000×; (**a**) Pyrolysis temperature: 600 °C; (**b**) Pyrolysis temperature: 800 °C; (**c**) Pyrolysis temperature: 1200 °C.

SEM images of untreated biochars of *P. vittata* and those chemically modified by FeCl_3_ and NaOH that were calcined at 600, 800, and 1200 °C are shown in Figure 1, Figure 2 and Figure 3, respectively.

The biochars at each pyrolysis temperature (Figure 1) showed an increase in the number of particles on the surface of the grain as the pyrolysis temperature increased. On the other hand, there was no significant difference in the surface structure of the biomass as the pyrolysis temperature was changed. Furthermore, in the case of chemical modifications of biochars, the FeCl_3_-treated sample (Figure 2) was found to have crystalline particles attached to the surface (Figure 2, right column). The adhesion of crystalline particles was observed on the chemically modified biochar surfaces of samples in the case of FeCl_3_ modification, probably caused by the precipitation of Fe_3_O_4_ or Fe on the surface, either directly or as complexes. Similar to the present study, chemical modification of biochars with FeCl_3_ was reported in a study of SEM-EDS, XRD, and FT-IR analysis of particles deposited on the surface, showing that Fe_3_O_4_ was deposited as an iron compound [26]. Fe^3+^ ions generated when FeCl_3_ is dissolved in water are known to be reduced to Fe in the presence of activated carbon in the aqueous phase [27], which explains the modification of Fe on the biochar surface. In the NaOH-treated sample (Figure 3), some of the layered structures on the biochar surface were detached. A porous structure caused by exfoliation of the surface structure was observed on the biochar surface treated with NaOH. Li et al. [20] reported that the specific surface area of biochar increased as a result of NaOH treatment. Therefore, it was considered that the surface of the biochar was eroded by the corrosive effect of NaOH, resulting in the exfoliation of the surface structure and the generation of pores. In addition, cracks on the biochar surface were observed in all the FeCl_3_- and NaOH-treated samples. 

Granular materials were identified on the surface of the non-treated biochar calcined at a temperature of 1200 °C, and they indicated that the functional groups on the surface of *P. vittata* leaves were decomposed and that basic surface oxides were formed. It has been reported that when Eucalyptus wood is carbonized at 1100 °C, basic elements such as Ca, which are not identified when carbonized at lower temperatures, precipitate on the surface [28]. Furthermore, Sasaki et al. reported that surface functional groups decompose and basic oxides are formed when carbon is heated in a vacuum or an inert airstream at temperatures greater than 1000 °C [29]. Leaves contain more chloroplasts than stems, and thus have a large number of sugars, which explains the presence of granular material in the biochars of leaves calcined at 1200 °C. 

In addition, the granular material identified in the leaf biochar calcined at 1200 °C disappeared after the NaOH treatment. This suggests that the granular material was composed of alkali-soluble substances. In addition, the cracks observed on the chemically modified biochar surface could be due to physical crushing by the agitator during the modification procedure. 

The specific surface areas of biochars at each pyrolysis temperature are listed in Table 2. The specific surface areas of the leaf biochar were 6.57 and 4.31 m^2^/g at 600 and 800 °C, respectively, with no significant difference. However, at 1200 °C, it was 34.54 m^2^/g, nearly five to seven times higher. The results showed that the specific surface area of the biochars increased with high-temperature pyrolysis. In addition, the specific surface areas of the FeCl_3_-modified leaf biochar were 160, 114, and 129 m^2^/g for pyrolysis temperatures of 600, 800, and 1200 °C, respectively, displaying a maximum increase of almost five times when compared to that before modification. The specific surface areas of the NaOH-modified biochar were 72.23, 31.92, and 65.38 m^2^/g at 600, 800, and 1200 °C, respectively. In the case of the NaOH-modified biochar, the highest specific surface area was observed at 600 °C. Therefore, the increase in the specific surface area of the biochar was higher with FeCl_3_ treatment than that of NaOH treatment. Considering the observation of the surface structure by SEM, it can be inferred that the specific surface area of the FeCl_3_-treated sample increased because of the adhesion of Fe to the surface and the formation of pores by the physical breaking of the biochar surface. In the case of the NaOH-modified biochars, exfoliation of the surface structure was also observed, which may be the reason for the increase in the specific surface area. Surface cracks were observed in both FeCl_3_- and NaOH-treated biochars, suggesting that the adhesion of Fe to the surface is a more significant factor in increasing the specific surface area.

Reported examples of biochar from other biological resources are rapeseed straw biochar calcined at 600 °C (19.13 m^2^/g) by Li et al. [20], cotton stalk biochar calcined at 600 °C (224 m^2^/g) by Zhang et al. [30], and rice straw biochar calcined at 800 °C (36.4 m^2^/g) by Basta et al. [31]. The specific surface area of FeCl_3_-modified biochar was reported by Cope et al. for rice husk at 550 °C (77.3 m^2^/g) [32] and Li et al. for rapeseed straw at 600 °C (6.82 m^2^/g) [20], as well as wheat stalks at 500 °C (72.68 m^2^/g) by Jiang et al. [26]. NaOH-modified sawdust at 700 °C (423.28 m^2^/g) was reported by Chen et al. [33], and rapeseed straw at 600 °C (43.18 m^2^/g) was reported by Li et al. [20]. The surface areas of the FeCl_3_-modified biochars of this study using *P. vittata* exceeded the values reported in previous studies. Previous studies have reported that chemical modification of FeCl_3_ results in a decrease in specific surface area when compared to that before the modification [20,26]. However, in the present study, the specific surface area was significantly increased. Different raw materials and different modification processes are possible reasons. In addition, the NaOH-modified biochars of this study had surface areas similar to those of the rapeseed straw biochar.

### 3.2. Evaluation of As and Cd Adsorption Capacities of P. vittata Biochar

**Figure 4:**
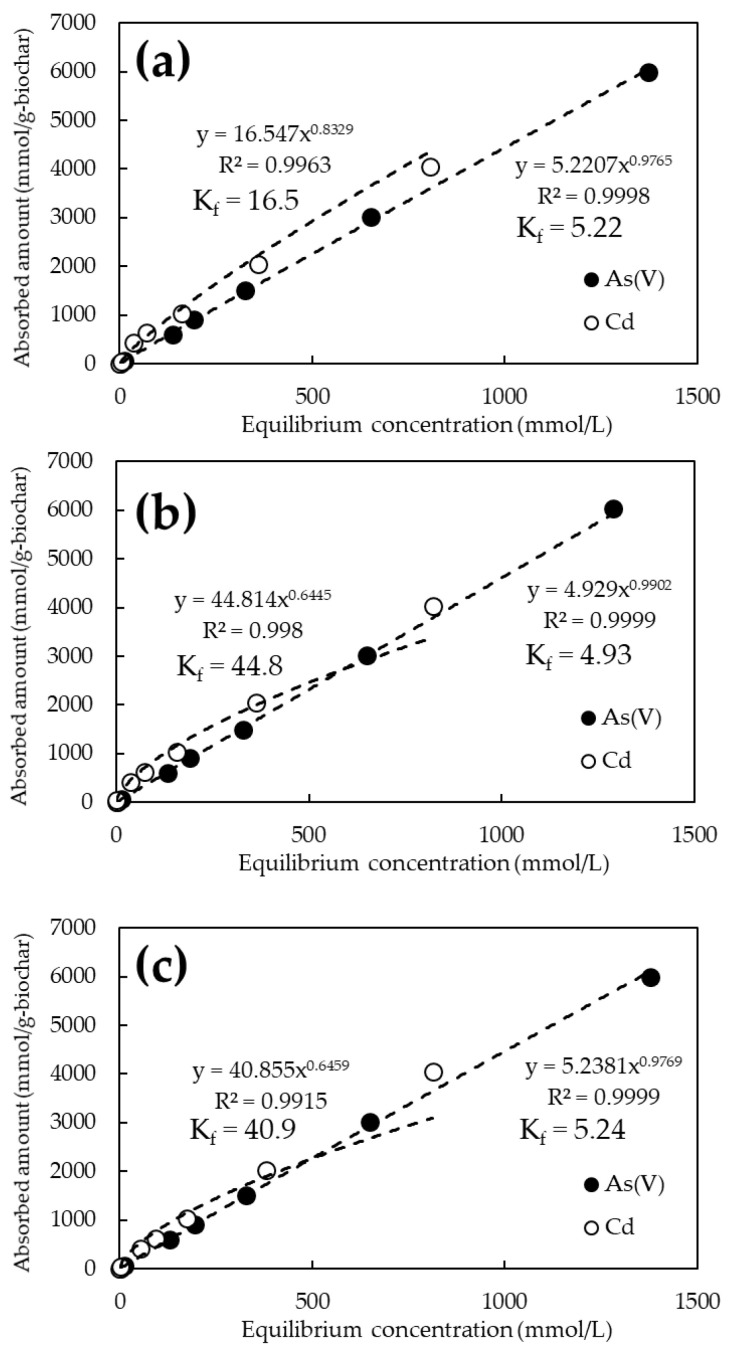
As(V) and Cd adsorption isotherms for biochars at various pyrolysis temperatures. (**a**) Pyrolysis temperature: 600 °C; (**b**) Pyrolysis temperature: 800 °C; (**c**) Pyrolysis temperature: 1200 °C.

Figure 4 shows the adsorption isotherms of As(V) and Cd for the unmodified biochars calcined at 600, 800, and 1200 °C. The pH of the tested solutions was 6.0 ± 0.2. Under these pH conditions, As(V) is considered to be ionized in solution as H_2_AsO_4_^−^ [34] and Cd as Cd^2+^ [35]. Hereafter, As(V) stands for H_2_AsO_4_^−^ and Cd for Cd^2+^ unless otherwise noted. Biochars calcined at all temperatures showed almost similar trends, adsorbing more Cd (Figure 4, open circle) than As(V) (Figure 4, closed circle). No clear difference in element adsorption behavior by pyrolysis temperature was observed. The quantity of As(V) and Cd adsorbed per unit weight of biochar at an initial concentration of 1000 mg/L was approximately 6000 and 4000 mmol/g-biochar at any pyrolysis temperature. The best adsorption values were 6031 mmol/g-biochar at 800 °C for As(V), and 4045 mmol/g-biochar at 600 °C for Cd. 

**Figure 5:**
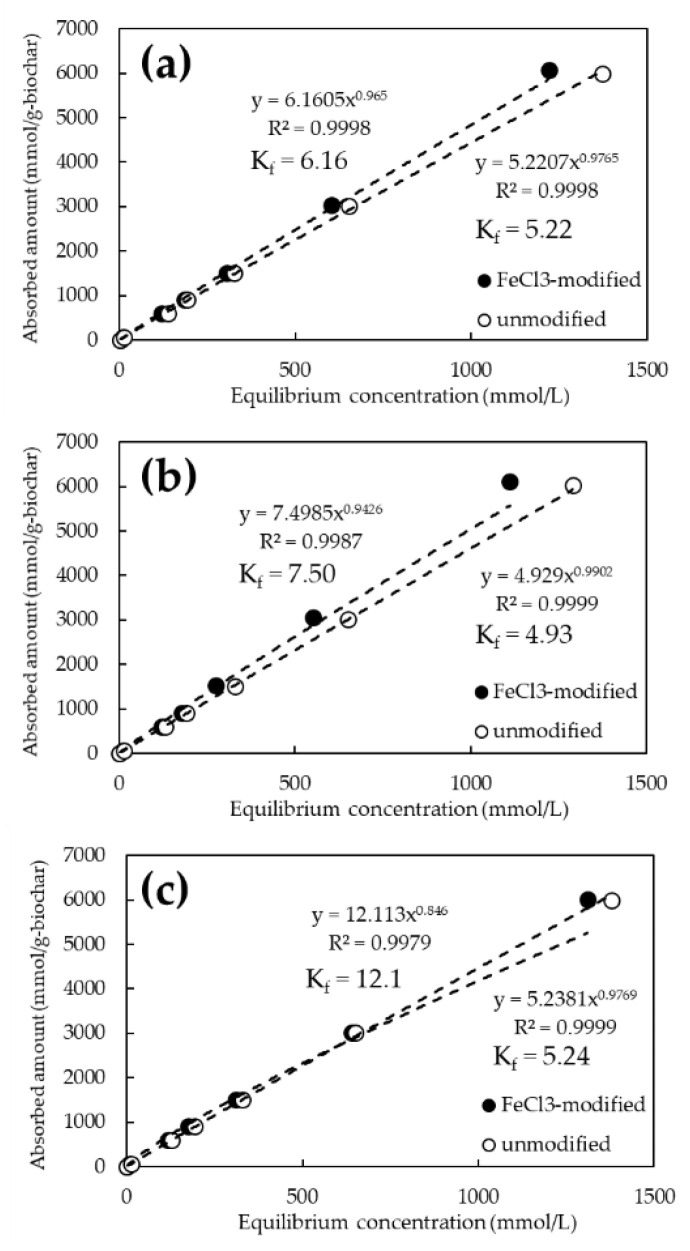
As(V) adsorption isotherms for FeCl_3_-modified and unmodified biochars at various pyrolysis temperatures. (**a**) Pyrolysis temperature: 600 °C; (**b**) Pyrolysis temperature: 800 °C; (**c**) Pyrolysis temperature: 1200 °C.

**Figure 6:**
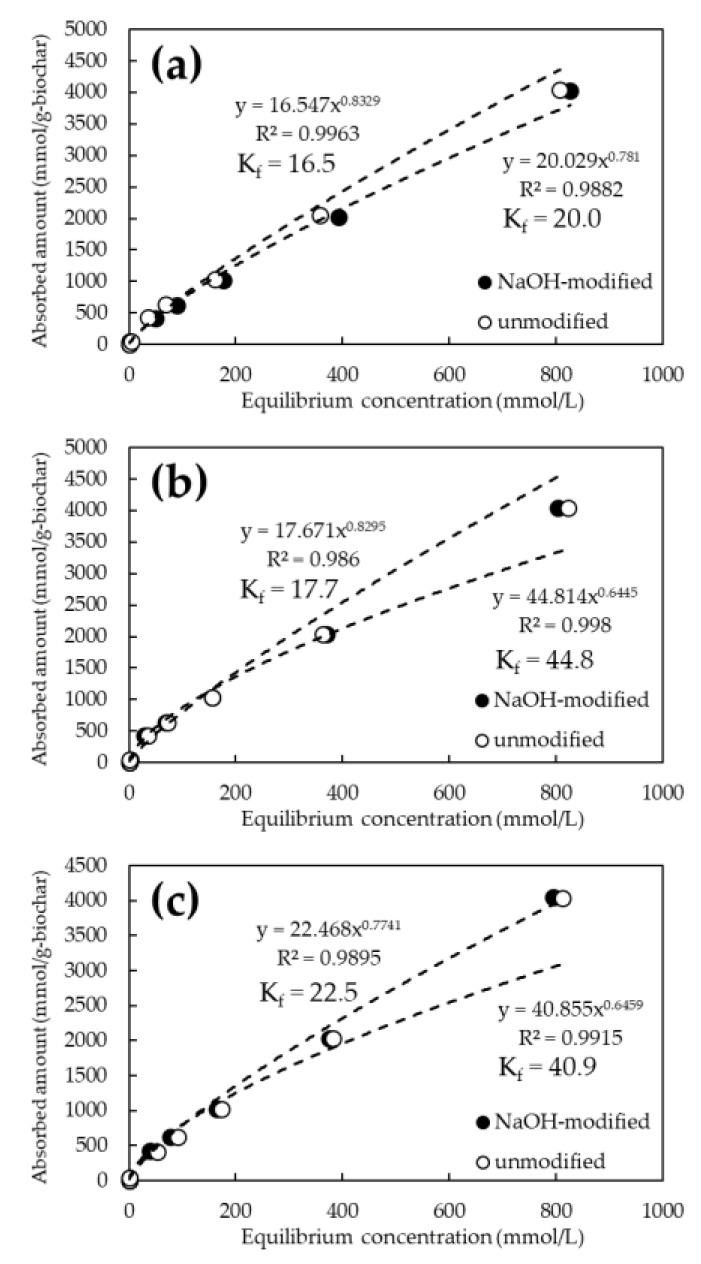
Cd adsorption isotherms for NaOH-modified and unmodified biochars at various pyrolysis temperatures. (**a**) Pyrolysis temperature: 600 °C; (**b**) Pyrolysis temperature: 800 °C; (**c**) Pyrolysis temperature 1200 °C.

Figure 5 and Figure 6 show the adsorption isotherms of As and Cd for the FeCl_3_ and NaOH-modified biochars, respectively. The chemical modification of the biochars resulted in a steeper slope of the approximate curve for As(V), confirming the improved adsorption performance (Figure 5). On the other hand, there was no significant difference in the adsorption performance of Cd between the NaOH-treated and untreated biochars (Figure 6). The best adsorption by biochar at the initial concentration of 1000 mg/L was 6120 mmol/g-biochar of As(V) at 800 °C for FeCl_3_ modification and 4051 mmol/g-biochar of Cd at 1200 °C for NaOH modification. Adsorption capacity increased by an average of approximately 2% in the case of FeCl_3_ modification. In particular, a performance increase of about 3.2% was observed at initial As(V) concentrations of 1 and 10 ppm. However, it was not significant in the case of NaOH modification.

The adsorption constant (K_f_) from the Freundlich model was obtained from the amount adsorbed on the biochar and the equilibrium concentration of the solution obtained from the adsorption test results. It is reported that the adsorption constant in the Freundlich model means the affinity to the target substance and adsorption capacity [36]. The K_f_ for As(V) ranged from 4.93 to 5.24 for the unmodified biochar, whereas it tended to increase to 6.16 to 12.1 for the FeCl_3_-modified biochar. In contrast, K_f_ for Cd ranged from 16.5 to 44.8 for unmodified biochar but tended to converge to a constant value of 17.7 to 22.5 for NaOH-modified biochar.

**Figure 7:**
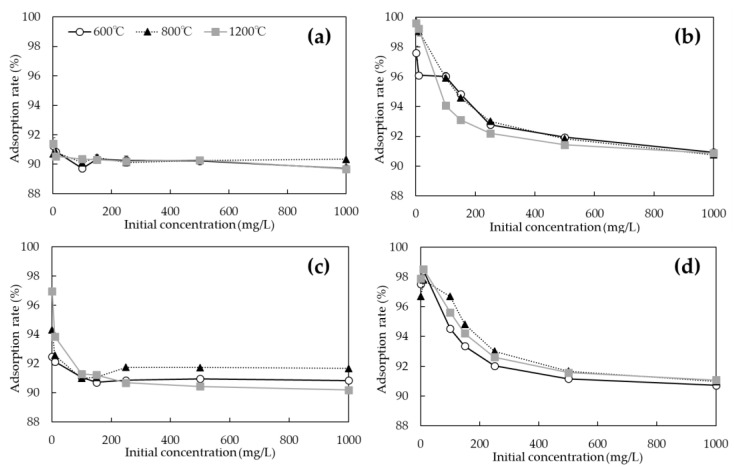
Adsorption rates of As(V) and Cd on biochars with and without chemical modification at each initial concentration. (**a**) As(V) adsorption of unmodified biochar; (**b**) Cd adsorption of unmodified biochar; (**c**) As(V) adsorption of FeCl_3_-modified biochar; (**d**) Cd adsorption of NaOH-modified biochar.

The adsorption rates of As(V) and Cd on the biochars with and without chemical modification at each initial concentration are shown in Figure 7. While the specific surface areas of unmodified biochars were much larger at 1200 °C than at 600 and 800 °C (Table 2), the adsorption rate of As(V) was only about 90% at any initial concentration (Figure 7a). However, the adsorption rate of the FeCl_3_-modified biochars improved at all initial concentrations at all pyrolysis temperatures (Figure 7c). In particular, it was shown that the adsorption rate in the low concentration range of 1 to 10 mg/L of initial concentration was improved when biochars were calcined at 800 and 1200 °C. Although some improvement or decrease in adsorption performance was observed for biochars modified with NaOH (Figure 7d), no significant change in the overall Cd adsorption properties was observed for biochars prepared at any pyrolysis temperature. The unmodified biochars showed a very high adsorption rate of about 97–98% under an initial Cd concentration of 1 to 10 mg/L (Figure 7b), from which the adsorption rate decreased as the initial concentration increased, and the adsorption rate remained at approximately 90% for biochars at any pyrolysis temperature at an initial concentration of 1000 mg/L. 

Table 3 shows the comparison of As(V) and Cd adsorption rates between commercially activated carbon (coconut biochar) and biochar prepared in this experiment (unmodified, FeCl_3_, and NaOH modified at a calculated 800 °C). In comparison with activated carbon, the adsorption rate of As(V) was inferior for untreated biochar at 1 and 10 mg/L. However, at other concentrations, the performance was comparable. In the case of FeCl_3_ modification, the adsorption rate of activated carbon was higher at an initial concentration of 1 mg/L, but at higher concentrations, FeCl_3_-modified biochar exceeded the adsorption rate of activated carbon. In terms of Cd adsorption rates, unmodified and NaOH-modified biochars, when compared to activated carbon, exceeded activated carbon adsorption rates at all initial concentrations. Therefore, the biochar made from *P. vittata* in this experiment was confirmed to have the same or higher performance when compared to commercially available activated carbon.

## 4. Discussion

The relationship between specific surface area and As(V) and Cd absorption is discussed. The specific surface area was evaluated using a monolayer adsorption model based on the Brunauer–Emmett–Teller (BET) equation with nitrogen adsorption, thus it is considered to be unaffected by the functional groups and chemical state of the biochar surface. In contrast, the adsorptions of As(V) and Cd were not affected by the specific surface area, suggesting some sites on the biochar surface specifically bind As(V) regardless of the pyrolysis temperature. 

On the other hand, Cd showed an extremely high adsorption capacity at low initial concentrations of 1 to 10 mg/L, while the adsorption rate decreased to 90% at an initial concentration of 1000 mg/L. This is because oxygen-containing functional groups, especially carboxyl (-COOH) groups, have high affinity and bind preferentially to cations such as Cd, but as the ratio of oxygen-containing functional groups to cations decreases with increasing concentration, the ratio of binding by surface π-electrons increases [37,38]. Therefore, this is a two-step adsorption mechanism. 

The specific surface area of the FeCl_3_-modified *P. vittata* biochar was larger than that of the unmodified biochar, and the As adsorption capacity improved. The specific surface area of the biochar calcined at 600 °C was the largest among the three types, but the increase in As adsorption capacity by FeCl_3_ modification was the highest for the biochar calcined at 1200 °C. However, the increase in the amount of As adsorbed by FeCl_3_-modification was the highest when the biochar calcined at 1200 °C was used as the raw material, probably because the Fe^3+^ ions were attracted to the π-electrons or oxygen-containing functional groups on the surface of this biochar than in the other two types [39]. The biochar calcined at 1200 °C before modification had a larger specific surface area than those calcined at 600 and 800 °C, and in this case, the adsorption by π-electrons is stronger because the oxygen-containing functional groups on the surface are fewer, due to the high-temperature pyrolysis. Therefore, it is suggested that the π-electrons of the biochar calcined at 1200 °C attracted more Fe^3+^ ions present in the aqueous solution [39], which increased the attachment of the reduced Fe or Fe_3_O_4_ particles to the biochar surface, or they caused the formation of complexes with oxygen-containing functional groups, increasing As(V) adsorption and specific surface area. 

In addition, the reason for the improved As(V) adsorption at low initial concentrations of 1 to 10 mg/L in the case of FeCl_3_ modification is that the biochars calcined at 800 and 1200 °C had a sufficient amount of Fe that was adsorbed on to their surfaces, which may have resulted in the formation of a high-affinity complex between Fe and As(V) [40]. In the biochar calcined at 600 °C, Fe was less modified than in the other two conditions, which may have caused the difference in the adsorption behavior after modification. On the other hand, it was confirmed that the adsorption performance tended to decrease at initial concentrations of above 100 mg/L when compared to that at lower concentrations because the complex formation by Fe has high affinity, but the number of adsorption sites is limited, and hence the adsorption rate may have decreased due to the increase in As(V) present in the surrounding area. 

Although the specific surface area of *P. vittata* biochar modified with NaOH was larger than that before modification, the Cd adsorption capacity improved only at the initial Cd concentration of 100–250 mg/L, probably because of the decrease in carboxyl groups (-COOH) associated with NaOH modification. It has been reported that the modification of biochar with NaOH results in the formation of lactones by dehydration condensation of the surface functional group COOH with the hydroxyl group (-OH) of NaOH and an increase in OH [20]. The *P. vittata* biochar calcined at 600 °C before modification had the highest amount of -COOH, and that calcined at 1200 °C was considered to have the strongest binding by π-electrons on the biochar surface. However, it was suggested that the NaOH modification reduced the surface functional group -COOH.

## 5. Conclusions

In this study, as a method for effective utilization of biomass containing toxic elements after phytoremediation, we investigated the reuse of an As hyperaccumulating plant, *P. vittata* biomass, as an environmental remediation material by generating biochars from it. SEM observation of the biochar surface showed that the elements contained in the biomass precipitated on the surface by melting when the pyrolysis temperature was high. The specific surface area was maximum for biochar under the condition of pyrolysis at 1200 °C. On the other hand, in the case of chemical modification, the specific surface area was maximum on the biochar fired at 600 °C for both FeCl_3_ and NaOH. Furthermore, in adsorption tests using As(V) and Cd, biochars showed high adsorption rates of over 90% for As(V) and Cd in the concentration range of 1 to 1000 mg/L. For Cd, in particular, the adsorption rate was over 93% at low initial concentrations of 1 and 10 mg/L. The adsorption constants (K_f_) for As(V) determined using the Freundlich model ranged from 4.93 to 5.24 for the unmodified biochar, whereas they tended to increase from 6.16 to 12.1 for the FeCl_3_-modified biochar. In contrast, K_f_ for Cd ranged from 16.5 to 44.8 for unmodified biochar but tended to converge to a constant value of 17.7 to 22.5 for NaOH-modified biochar. In the case of chemical modified biochar, the As adsorption rate was improved by FeCl_3_ treatment. However, the adsorption capacity for Cd was not significantly affected by the NaOH treatment. In conclusion, it was found that after phytoremediation using *P. vittata* biomass, it can be effectively used as an environmental purification material by conversion to biochar. Furthermore, chemical modification with FeCl_3_ improves the biochar’s adsorption performance.

## Figures and Tables

**Table 1:** Summary of experimental conditions for As(V) and Cd adsorption experiments.

Modification	—			FeCl_3_			NaOH		
**Pyrolysis** **Temperature**	**600 °C**	**800 °C**	**1200 °C**	**600 °C**	**800 °C**	**1200 °C**	**600 °C**	**800 °C**	**1200 °C**
As(V)	○	○	○	○	○	○			
Cd	○	○	○				○	○	○

A circle in the table indicates that it was implemented. All experiments were conducted with 3 independent sample replications.

**Table 2:** Specific surface area (m^2^/g) of biochars at each pyrolysis temperature and after chemical modification.

	Pyrolysis Temperature		
**Treatment**	**600 °C**	**800 °C**	**1200 °C**
Unmodified	6.57 ± 0.22	4.31 ± 0.13	34.54 ± 0.92
FeCl_3_-modified	160.83 ± 2.54	114.78 ± 1.83	129.35 ± 1.98
NaOH-modified	72.23 ± 1.65	31.92 ± 0.85	65.38 ± 1.26

**Table 3:** Comparison of As(V) and Cd adsorption rates between commercially activated carbon (coconut biochar) and biochar prepared in this experiment (unmodified, FeCl_3_ and NaOH modified at a calculated 800 °C).

		Absorption Rate (%)						
	**Initial Concentration (mg/L)**	**1**	**10**	**100**	**150**	**250**	**500**	**1000**
Activate carbon	As(V)	99.31 ± 0.01	92.72 ± 0.37	90.15 ± 0.31	90.14 ± 0.20	90.16 ± 0.16	89.82 ± 0.14	89.68 ± 0.20
	Cd	93.84 ± 0.05	92.52 ± 0.14	91.38 ± 0.01	91.01 ± 0.32	90.79 ± 0.23	90.64 ± 0.13	90.33 ± 0.32
Biochar	As(V)	90.72 ± 0.23	90.80 ± 0.10	90.07 ± 0.14	90.52 ± 0.18	90.13 ± 0.25	90.26 ± 0.09	90.32 ± 0.11
	Cd	99.71 ± 0.04	99.13 ± 0.32	95.97 ± 0.22	94.64 ± 0.13	92.95 ± 0.09	91.80 ± 0.07	90.79 ± 0.11
FeCl_3_-modified	As(V)	94.34 ± 0.33	92.60 ± 0.09	91.01 ± 0.03	91.11 ± 0.22	91.82 ± 0.27	91.67 ± 0.08	91.66 ± 0.11
NaOH-modified	Cd	96.73 ± 0.02	97.82 ± 0.01	96.73 ± 0.20	94.81 ± 0.19	93.01 ± 0.08	91.67 ± 0.07	91.04 ± 0.09

± means SD. All experiments were conducted with 3 independent sample replications.

## Data Availability

The data presented in this study are available on request from the corresponding author upon reasonable request.

## References

[1] Duker A., Carranza E., Hale M. (2005). Arsenic geochemistry and health. Environ. Int..

[2] Hans Wedepohl K. (1995). The composition of the continental crust. Geochim. Cosmochim. Acta.

[3] Argos M., Kalra T., Rathouz P.J., Chen Y., Pierce B., Parvez F., Islam T., Ahmed A., Rakibuz-Zaman M., Hasan R. (2010). Arsenic exposure from drinking water, and all-cause and chronic-disease mortalities in Bangladesh (HEALS): A prospective cohort study. Lancet.

[4] Bagchi S. (2007). Arsenic threat reaching global dimensions. Can. Med. Assoc. J..

[5] Bernhoft R.A. (2013). Cadmium Toxicity and Treatment. Sci. World J..

[6] Zwicker R., Promsawad A., Zwicker B.M., Laoharojanaphand S. (2010). Cadmium Content of Commercial and Contaminated Rice, Oryza sativa, in Thailand and Potential Health Implications. Bull. Environ. Contam. Toxicol..

[7] Wang P., Chen H., Kopittke P.M., Zhao F.J. (2019). Cadmium contamination in agricultural soils of China and the impact on food safety. Environ. Pollut..

[8] Matović V., Buha A., Bulat Z., Đukić-Ćosić D. (2011). Cadmium Toxicity Revisited: Focus on Oxidative Stress Induction and Interactions with Zinc and Magnesium. Arch. Ind. Hyg. Toxicol..

[9] Pilon-Smits E. (2005). Phytoremediation. Annu. Rev. Plant Biol..

[10] Verbruggen N., Hermans C., Schat H. (2009). Molecular mechanisms of metal hyperaccumulation in plants. New Phytol..

[11] Ma L.Q., Komar K.M., Tu C., Zhang W., Cai Y., Kennelley E.D. (2001). A fern that hyperaccumulates arsenic. Nature.

[12] Nedelkoska T.V., Doran P.M. (2000). Hyperaccumulation of cadmium by hairy roots of *Thlaspi caerulescens*. Biotechnol. Bioeng..

[13] Ingle R.A., Smith J.A.C., Sweetlove L.J. (2005). Responses to Nickel in the Proteome of the Hyperaccumulator Plant *Alyssum lesbiacum*. BioMetals.

[14] Salt D.E., Prince R.C., Pickering I.J., Raskin I. (1995). Mechanisms of Cadmium Mobility and Accumulation in Indian Mustard. Plant Physiol..

[15] Tognacchini A., Rosenkranz T., van der Ent A., Machinet G.E., Echevarria G., Puschenreiter M. (2020). Nickel phytomining from industrial wastes: Growing nickel hyperaccumulator plants on galvanic sludges. J. Environ. Manag..

[16] Lehmann J., Joseph S. (2012). Biochar for Environmental Management.

[17] Keiluweit M., Nico P.S., Johnson M.G., Kleber M. (2010). Dynamic Molecular Structure of Plant Biomass-Derived Black Carbon (Biochar). Environ. Sci. Technol..

[18] Lee M.E., Park J.H., Chung J.W. (2017). Adsorption of Pb(II) and Cu(II) by ginkgo-leaf-derived biochar produced under various carbonization temperatures and times. Int. J. Environ. Res. Public Health.

[19] Samsuri A.W., Sadegh-Zadeh F., Seh-Bardan B.J. (2013). Adsorption of As(III) and As(V) by Fe coated biochars and biochars produced from empty fruit bunch and rice husk. J. Environ. Chem. Eng..

[20] Li B., Yang L., Wang C., Zhang Q., Liu Q., Li Y., Xuao R. (2017). Adsorption of Cd(II) from aqueous solutions by rape straw biochar derived from different modification processes. Chemosphere.

[21] Datta R., Das P., Tappero R., Punamiya P., Sahi S., Feng H., Kiiskila J., Sarkar D. (2017). Evidence for exocellular Arsenic in Fronds of Pteris vittata. Sci. Rep..

[22] Sugawara K., Kitajima N., Suzuki S. (2018). Analysis of essential elements behavior accompanying with arsenic accumulation in arsenic hyper accumulator and non-accumulating plants by multivariate analysis. Environ. Biotechnol..

[23] Guillory J.K. (2007). The Merck Index: An Encyclopedia of Chemicals, Drugs, and Biologicals Edited by Maryadele J. O’Neil, Patricia E. Heckelman, Cherie B. Koch, and Kristin J. Roman. Merck, John Wiley & Sons, Inc., Hoboken, NJ. 2006. xiv + 2564 pp. 18 × 26 cm. ISBN-13 978-0-911910-001. $125.00. J. Med. Chem..

[24] Yang C., Ho Y.N., Inoue C., Chien M.F. (2020). Long-term effectiveness of microbe-assisted arsenic phytoremediation by *Pteris vittata* in field trials. Sci. Total Environ..

[25] Brunauer S., Emmett P.H., Teller E. (1938). Adsorption of Gases in Multimolecular Layers. J. Am. Chem. Soc..

[26] Jiang Y., Dai M., Yang F., Ali I., Naz I., Peng C. (2022). Remediation of Chromium (VI) from Groundwater by Metal-Based Biochar under Anaerobic Conditions. Water.

[27] Shirakashi T. (1993). Reduction Reaction of Fe^3+^ Ion by Activated Carbon. Nippon Kagaku Kaishi.

[28] Choo W., Hayashi T., Ohashi C., Sugawara K., Ito T., Suzuki S., Kato S., Kojima T. (2018). Formulation of gasification rate of char from *Eucalyptus camaldulensis* grown in arid land on Western Australia. J. Arid. Land Stud..

[29] Sasaki M., Tamai H., Yoshida T., Yasuda H. (1998). Dye Adsorption on Mesoporous Activated Carbon Fiber Obtained from Pitc Containing Yttrium Complex and tha Acid Treatment Effects. Tanso.

[30] Zhang X., Zhang S., Yang H., Feng Y., Chen Y., Wang X., Chen H. (2014). Nitrogen enriched biochar modified by high temperature CO_2_-ammonia treatment: Characterization and adsorption of CO_2_. Chem. Eng. J..

[31] Basta A.H., Fierro V., El-Saied H., Celzard A. (2009). 2-Steps KOH activation of rice straw: An efficient method for preparing high-performance activated carbons. Bioresour. Technol..

[32] Cope C.O., Webster D.S., Sabatini D.A. (2014). Arsenate adsorption onto iron oxide amended rice husk char. Sci. Total Environ..

[33] Chen Z., Jing Y., Wang Y., Meng X., Zhang C., Chen Z., Zhou J., Oiu R., Zhang X. (2020). Enhanced removal of aqueous Cd(II) by a biochar derived from salt-sealing pyrolysis coupled with NaOH treatment. Appl. Surf. Sci..

[34] Baig S.A., Sheng T., Hu Y., Xu J., Xu X. (2015). Arsenic Removal from Natural Water Using Low Cost Granulated Adsorbents: A Review. Clean Soil Air Water.

[35] Yang T., Sheng L., Wang Y., Wyckoff K.N., He C., He Q. (2018). Characteristics of Cadmium Sorption by Heat-Activated Red Mud in Aqueous Solution. Sci. Rep..

[36] Kano F., Abe I., Kamaya H., Ueda I. (2000). Fractal model for adsorption on activated carbon surfaces: Langmuir and Freundlich adsorption. Surf. Sci..

[37] Salame I.I., Bandosz T.J. (2003). Role of surface chemistry in adsorption of phenol on activated carbons. J. Colloid Interface Sci..

[38] Radovic L.R., Silva I.F., Ume J.I., Menéndez J.A., Leon C.A., Leon Y., Scaroni A.W. (1997). An experimental and theoretical study of the adsorption of aromatics possessing electron-withdrawing and electron-donating functional groups by chemically modified activated carbons. Carbon.

[39] Kato Y., Machida M., Tatsumoto H. (2007). Influence of Surface Functional Groups and Solvent on Adsorption of Dissolved Aromatics by Activated Carbon. J. Environ. Chem..

[40] Couture R.M., Rose J., Kumar N., Mitchell K., Wallschläger D., Van Cappellen P. (2013). Sorption of arsenite, arsenate, and thioarsenates to iron oxides and iron sulfides: A kinetic and spectroscopic investigation. Environ. Sci. Technol..

